# Small bowel involvement documented by capsule endoscopy in Churg-Strauss syndrome

**DOI:** 10.11604/pamj.2015.22.41.7810

**Published:** 2015-09-17

**Authors:** Birane Beye, Gilles Lesur, Pierre Claude, Lionel Martzolf, Pierre Kieffer, Daniel Sondag

**Affiliations:** 1 Service de Gastroentérologie, Hôpital Ambroise Paré, 9 avenue Charles de Gaulle, 92100 Boulogne, France; 2 UFR 2S, Université Gaston Berger, St-Louis, Sénégal; 3 Service de Gastroentérologie, Centre Hospitalier, 20 Avenue Réné Laennec, 68100 Mulhouse, France; 4 Service de Médecine Interne, Centre Hospitalier, 20 Avenue Réné Laennec, 68100 Mulhouse, France

**Keywords:** Churg-Strauss Syndrome, small intestine involvement, video capsule endoscopy

## Abstract

Churg-Strauss syndrome is a small and medium vessel vasculitis and is also known as allergic granulomatous angiitis. Gastrointestinal involvement is common in patients with Churg-Strauss syndrome (20-50%). The most common symptoms are abdominal pain, diarrhoea and occasionally gastrointestinal bleeding and perforation. We present a case of Churg-Strauss syndrome with small bowel lesions documented by video capsule endoscopy.

## Introduction

Churg-Strauss syndrome (CSS) is a systemic and pulmonary vasculitis characterized by the combination of severe late-onset asthma, tissue and blood eosinophilia (greater than 1.5 G/L). It affects small and medium vessels, with segmental and transmural lesions, extravascular tissue infiltrates by eosinophil granulocytes, and granulomas with epithelioid and giant polynuclear cells [1]. Gastrointestinal events frequency is estimated between 20 and 50% of cases [2]. The most common symptoms are abdominal pain, diarrhoea and gastrointestinal bleeding [3]. Small bowel ulcer diagnosed by endoscopy is exceptionally reported in the literature [2]. We report a new case of Churg-Strauss syndrome presenting with small bowel lesions documented by capsule endoscopy.

## Patient and observation

**Figure 1:**
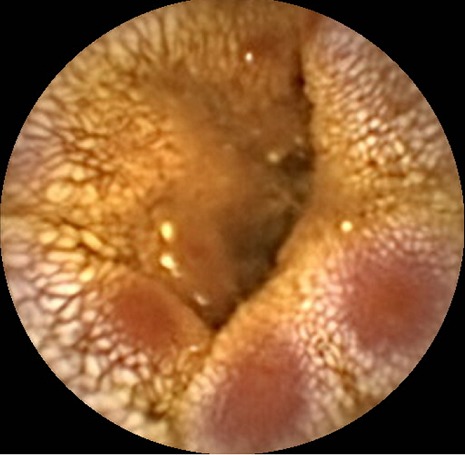
Submucosal edema with lymphangiectasias and erythematous sites on top of some jejunal valves

**Figure 2:**
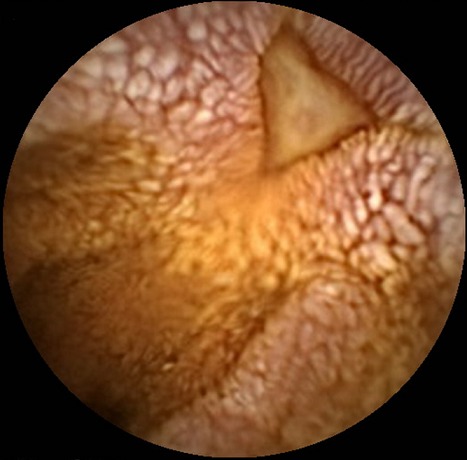
Geometric ulcer (triangular)

A 30-year-old man was assessed at our department for severe abdominal pain. His past medical history consisted of multiple allergies (pollen, dust mites, dog and cat hair) and a short three-day hospital stay for an exacerbation of asthma managed with oral corticosteroid treatment and bronchodilators. On admission, his temperature was 38°C and blood pressure 120/60 mmHg. Clinical examination of the abdomen and the digital rectal exam were normal. Initial investigations showed an elevated white blood cell count (15600/mm^3^) with marked eosinophilia (8700/mm^3^) and a high CRP level at 300mg/L (N < 5) but normal renal and hepatic function. Blood and urine cultures, as well as parasite detection tests, were negative. Antineutrophil cytoplasmic antibodies (ANCA), desoxyribonucleic acid (DNA) antibody, anti-nuclear antigen were negative. Cryoglobulin and serologic markers for human immunodeficiency virus (HIV), hepatitis B virus (HBV) and hepatitis C virus (HCV) infection were negative. Upper gastrointestinal endoscopy pushed into the proximal jejunum showed an irregular aspect of the jejunal mucosa, without ulcers, corresponding to sub-acute inflammatory lesions, rich in eosinophils, without granulomas or signs of necrotizing vasculitis at histopathological biopsy analysis. Colonoscopy was normal and biopsies were not taken. Due to the persistence of abdominal pain, capsule endoscopy was performed, revealing submucosal edema with numerous lymphangiectasias and erythematous sites on top of some jejunal valves (Figure 1), as well as large ulcers of various shapes, occasionally geometric (Figure 2). The patient was re-admitted after a few weeks, in the dermatology ward for urticaria and polyarthritis. Treatment with prednisolone (40 mg/day) and azathioprine (2mg/kg/day) was introduced, with good clinical response. A few weeks later, he returned to the emergency department because of a generalized epileptic crisis with cerebral magnetic resonance imaging (MRI) findings suggestive of a likely vascular origin. In addition, heart, kidney and ENT (ear, nose and throat) abnormalities were documented. Taking into account the above clinical and biological evidence, and in light of the capsule endoscopy-demonstrated ulcers, a systemic disease was suspected, leading to a deltoid muscle biopsy which showed necrotizing vasculitis of small vessels with eosinophilia. The combination of asthma, eosinophilia more than 8000/mm^3^ and necrotizing vasculitis with eosinophilic infiltration of small vessels permitted to establish the diagnosis of CSS. Treatment with cyclophosphamide was started, with inflammatory syndrome and eosinophilia decrease. The initial response was good in terms of clinical symptoms and biological markers control.

## Discussion

The CSS diagnosis has been retained in our case in view of the combination of asthma, blood eosinophilia, inflammatory syndrome, pulmonary infiltrates and extrapulmonary events. Five of the six ACR (American College of Rheumatology) diagnostic criteria (asthma, blood eosinophilia > 10%, peripheral neuropathy, pulmonary infiltrates, abnormal maxillary sinus and extravascular tissue eosinophilia) were present in our patient [1]. Presence of at least four of these criteria allows the diagnosis of CSS, with a sensitivity of 85% and a specificity of 99.7%. Gastrointestinal tract involvement represents the fourth leading cause of death in cases of severe disease after cardiac, neurological and kidney manifestations [4]. Gastrointestinal symptoms during CSS occur in 54% of patients on average, marked, however, by significant variations of up to 92%. The most common symptoms are abdominal pain, representing 59% [3], followed by nausea, vomiting and diarrhoea. In our patient, gastrointestinal symptoms were the first extrapulmonary manifestation and the revelation mode of CSS, a fact in itself rare [5]. This digestive-onset form may be particularly compromising and a poor prognostic factor according to some authors [2], encompassing many dangerous complications perforation [6, 7], ischemia [4, 6], bleeding [5, 8] or obstruction [4], responsible for 30% of deaths during CSS. In our case report, the intestinal disease was analysed using the technique of capsule endoscopy, so far reported only once in the literature [2].

## Conclusion

Gastrointestinal events during Churg and Strauss syndrome are reported in one third of patients on average. Most macroscopic involvement are identified by conventional endoscopy, laparotomy or at autopsy. Ulcerations of the small intestine are very few described, and prevalence underestimated, because their exploration is often incomplete. We must therefore think of capsule endoscopy as the need for their evidence is real, because representing part of bad prognostic factors, especially when they are severe, forcing an emergency induction therapy.

## References

[1] Noth I, Strek ME, Leef AR (2003). Churg-Strauss syndrome. Lancet..

[2] Sanchez R, Aparicio JR, Baeza T, Calero Y (2006). Capsule endoscopy diagnosis of intestinal involvement in a patient with Churg-Strauss syndrome. Gastrointest Endosc..

[3] Lanham JG, Elkon K, Pusey C, Hughes G (1984). Systemic vasculitis with asthma and eosinophilia: a clinical approach to the Churg-Strauss Syndrome. Medicine..

[4] Pagnoux C, Mahr A, Guillevin L (2003). Manifestations abdominales et digestives au cours des vascularites systémiques. Ann Med Intern..

[5] Fraioli P, Barberis M, Rizzato G (1994). Gastrointestinal presentation of Churg-Strauss syndrome. Sarcoidosis..

[6] Murakami S, Misumi M, Sakata H, Hirayama R, Kubojima Y, Nomura K, Ban S (2004). Churg-Strauss syndrome manifesting as perforation of the small intestine: report of a case. Surg Today..

[7] Sharma MC, Rajni S, Sidhu BS (1996). Perforation of small intestine caused by Churg-Strauss syndrome. J Clin Gastroenterol..

[8] Kazuhiko H, Yasushi H, Hiroyuki T, Youshin A, Yukisato k, Masako K (2005). Ileal ulcers and cytomegalovirus infection in case of Churg-Strauss syndrome. Arch Pathol Lab Med.

